# Tanycyte Gene Expression Dynamics in the Regulation of Energy Homeostasis

**DOI:** 10.3389/fendo.2019.00286

**Published:** 2019-05-07

**Authors:** Fanny Langlet

**Affiliations:** Center for Integrative Genomics, University of Lausanne, Lausanne, Switzerland

**Keywords:** tanycyte, metabolic sensing, gene expression, energy balance, hypothalamus

## Abstract

Animal survival relies on a constant balance between energy supply and energy expenditure, which is controlled by several neuroendocrine functions that integrate metabolic information and adapt the response of the organism to physiological demands. Polarized ependymoglial cells lining the floor of the third ventricle and sending a single process within metabolic hypothalamic parenchyma, tanycytes are henceforth described as key components of the hypothalamic neural network controlling energy balance. Their strategic position and peculiar properties convey them diverse physiological functions ranging from blood/brain traffic controllers, metabolic modulators, and neural stem/progenitor cells. At the molecular level, these functions rely on an accurate regulation of gene expression. Indeed, tanycytes are characterized by their own molecular signature which is mostly associated to their diverse physiological functions, and the detection of variations in nutrient/hormone levels leads to an adequate modulation of genetic profile in order to ensure energy homeostasis. The aim of this review is to summarize recent knowledge on the nutritional control of tanycyte gene expression.

## Introduction

Living organisms require an adequate balance between energy supply and energy expenditure to maintain cell and organ functions. While all cells are able to sense systemic cues of the immediate environment in order to maintain energetic and cellular stability, the central nervous system is often considered as the conductor orchestrating the maintenance of energy homeostasis by sensing the global metabolic state and responding via efferent regulatory signals (1).

**Figure 1:**
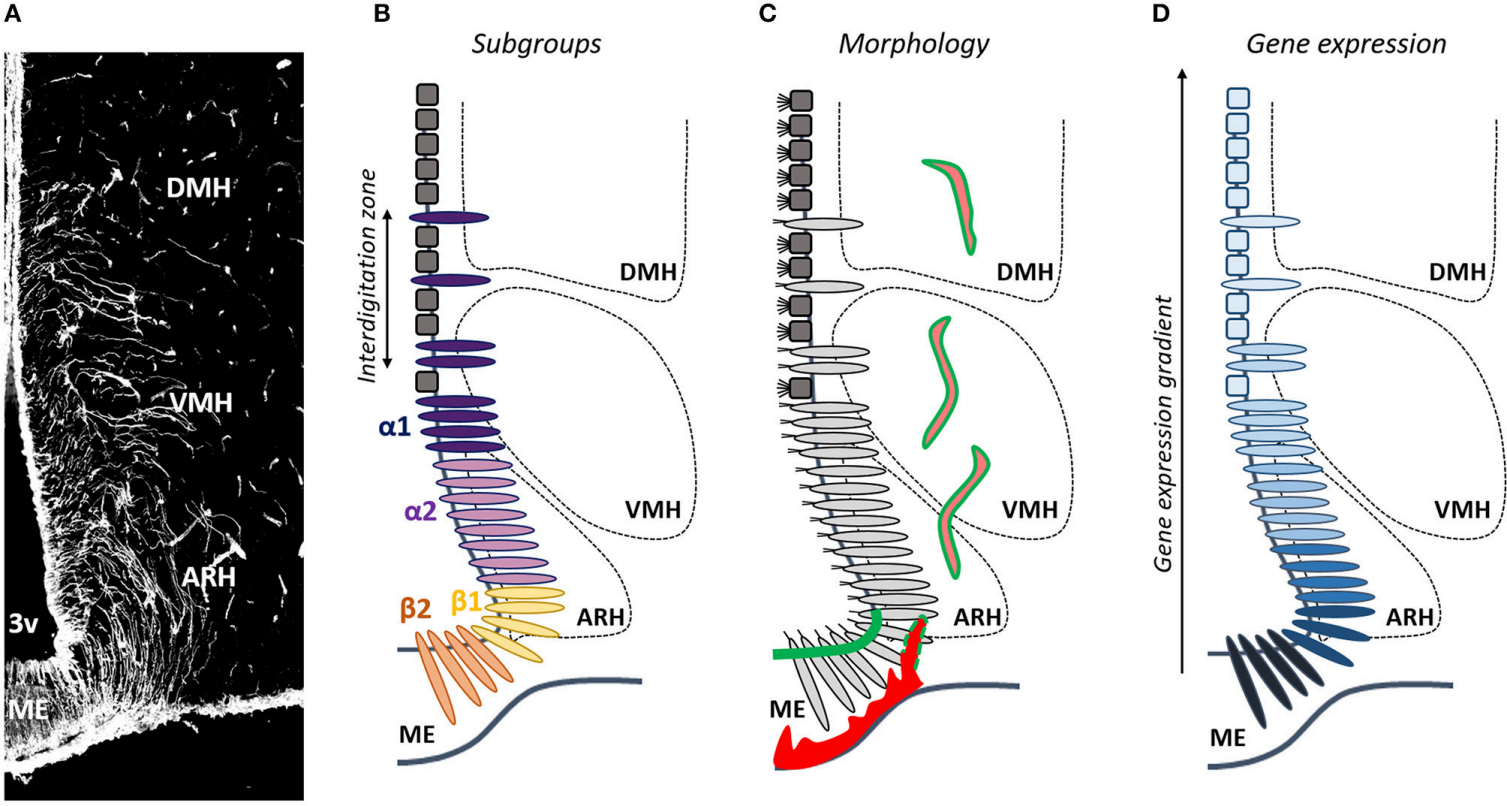
Is tanycyte subtype classification obsolete? **(A)** Tanycytes are polarized ependymoglial cells lining the basal part of the third ventricle (3V), visualized by vimentin immunostaining (white). **(B)** Tanycytes have been classified in four subtypes (β1, β2, α1, and α2). **(C)** Tanycytes (light gray) are morphologically distinguished from classical multiciliated cuboidal ependymal cells (dark gray). They send a single long radial process into the mediobasal hypothalamus including the median eminence (ME), the arcuate nucleus (ARH), the ventromedial nucleus (VMH), and the dorsomedial nucleus (DMH). Tanycytes lining the ME and vmARH are uniciliated cells, contact fenestrated vessels (red) and carry blood/brain barrier (green), whereas tanycytes lining the dmARH, the VMH and the DMH are biciliated cells, and contact neurons and blood/brain barrier vessels (pink/green). In the vmARH, vessels are permeable or not according to the energy status of the individual (red/green dashes). **(D)** Many genes exhibite a gradient, rather than a clear-cut distribution across tanycyte subpopulations.

A large number of brain regions have been recognized to play a role in metabolic homeostasis, but neuronal networks mainly converge to the hypothalamus, which contains numerous neural cells that influence feeding and energy expenditure (1, 2). Among these cells, tanycytes have been described as a component of the hypothalamic neural network controlling energy balance (3–6). Tanycytes are special elongated and polarized ependymoglial cells that line the lateral walls and the floor of the third ventricle (Figure 1) (7–9). They are morphologically distinguished from more dorsally-located classical cuboidal ependymal cells by the absence of beating cilia that drive the flow of cerebrospinal fluid (CSF), and by the presence of a single long radial process sent into the mediobasal hypothalamus including the median eminence (ME) and hypothalamic nuclei involved in the regulation of energy balance (Figure 1) (8). Due to their strategic position in contact with 1—the CSF at their apical surface, 2—fenestrated blood capillaries in the ME, and 3—blood-brain-barrier vessels and/or neurons that regulate appetite/energy expenditure in the hypothalamic parenchyma (Figure 1), tanycytes are henceforth considered as crucial components of energy homeostasis regulation. Indeed, their versatile functions include the dynamic regulation of blood-brain and blood-CSF exchanges (10), the shuttling of circulating metabolic signals to hypothalamic neurons (11, 12), the detection of the metabolic state of the animal (10, 13–15), and neural stem cell properties (16, 17). Moreover, they are able to adapt the above-mentioned functions to the physiological state of the animal (10, 11) which allow them to constitute a key gear component in the hypothalamic regulation of energy balance.

To regulate energy balance, one key process is the coordination between gene expression and metabolic status of the individual (18, 19). Indeed, molecular mechanisms relay environmental metabolic cues such as nutrient availability and/or hormone levels to the appropriate gene expression response, that will ensure the appropriate cellular function and thus the latter's regulation of energy balance. Although poorly described, these processes also occur in tanycytes during energy imbalance in order to adapt their functions to the metabolic state. This review will focus on the regulation of gene expression in tanycytes necessary for their function and plasticity in the metabolic hypothalamus. It will first define each tanycyte subtypes associating their molecular signature to their specific functions. Secondly, I will provide our current understanding about the effects of nutrition on tanycyte gene expression and its consequent impact on tanycyte function. Finally, the potential molecular mechanisms leading to these modulations will be discussed.

## Molecular Insights into Tanycyte Classification and Metabolic Function

### Tanycyte Classification

Tanycytes do not constitute a homogeneous cell population (20–22); to the contrary, they show a complex heterogeneity which is not fully comprehended yet. Depending on their dorsoventral location along the third ventricle, the different hypothalamic nuclei where their processes are sent, and their morphological, structural, genetic and functional properties (20), tanycytes have been classified in four subtypes (β*1*, β*2*, α*1*, and α*2*) (Figure 1). β*2* tanycytes line the floor of the third ventricle in the ME of the hypothalamus, and contact the perivascular space facing the hypothalamo-hypophysial portal system—characterized by a fenestrated endothelium—together with neuroendocrine secretory axon terminals. β*1* tanycytes line the lateral evaginations of the infundibular recess and the area of the ventromedial arcuate nucleus (vmARH), and contact *en passant* periventricular vessels before continuing into the parenchyma up to the perivascular space of lateral ME fenestrated vessels or the pial surface of the brain. α*2* tanycytes line the area of the dorsomedial arcuate nucleus (dmARH), whereas α*1* tanycytes line the ventromedial (VMH) and dorsomedial nuclei (DMH). α tanycyte processes are sent into the brain parenchyma where they contact blood-brain barrier (BBB) vessels and/or neurons (20).

### Tanycyte Genetic Profiles Are Associated to Specific Metabolic Functions

While some genes are commonly expressed in the entire tanycyte population (e.g., *Ppp1r1b, Vim, Rax, Dio2, Slc16a2*) (23, 24), each tanycyte subtype also exhibits its own molecular signature which is mostly associated to their various physiological functions in the regulation of energy homeostasis.

Hence, *β* tanycytes are described as component of blood-brain interface (8, 25, 26) able to dynamically control the access of nutrients and hormones to the brain (10), as well as the secretion of neuropeptides into the hypothalamo-hypophysial vascular system in the ME (3, 27, 28); whereas α-tanycytes are described as modulators of neuronal activity (29–31). Both α- and β-tanycytes are also considered as chemosensitive cells (4, 6) and diet-responsive adult neural stem cells (32). Although the molecular mechanisms underlying these various functions remain largely unknown, numerous genes have been associated to them in the literature and/or using publicly available gene expression databases (i.e., Allen brain atlas) (4, 5, 20, 23, 24, 33). Therefore, *β* tanycytes in direct contact with fenestrated capillaries are the only subtype to express tight junction protein Claudin1 (*Cldn1*) (8–10), necessary to ensure a tight blood-CSF barrier, and the vascular endothelial growth factor (*Vegf* ) (10), necessary to ensure the permeability of ME vessels. In another hand, they specifically express N-Cadherin (*Cdh2*) and Caveolin-1 (*Cav1*) (34), which are implicated in endocytosis/transcytosis, and/or recycling of cell surface receptors for the regulation of cell signaling. Still related to their transporter properties, GLUT1–known as facilitated glucose transporter member 1 (*Slc2a1*)–is expressed in β*1* tanycytes and, to a lesser extent, in β*2* tanycytes (34–36). *β* tanycytes have also restricted expression of neural stem markers such as *Sox2* (16, 17, 37, 38)*, Fgf-10* (17, 39, 40), *Blbp* (17), and *Musashi1* (17), as well as several growth factor receptor genes, such as *Fgfr1* (38, 41) and *Cntfr* (42), consistent with their stem/ progenitor cell function. In contrast, the gene markers of *α* tanycytes are more similar to non-tanycyte ependymal cells than *β* tanycytes. In particular, *α* tanycytes making contact with BBB capillaries and/or neurons express GLAST (*Slc1a3*) (17, 43), MCT1 (*Slc16a1*) and MCT4 (*Slc16a4*) (29) involved in the recapture of glutamate and lactate transport, respectively, necessary for the modulation of neuronal activity. They also express connexin 43 (*Gja1*), a component of gap junction which allow intercellular communications between adjacent cells (44–46). *α* tanycytes also express *Fgf18* (40) and *Prss56* (47), which have been associated to their stem/progenitor cell function.

### Towards a New Tanycyte Classification

Although helpful, this classification in *α* vs. *β* tanycytes is a too simplistic generalization. Indeed, recent advances in our understanding of tanycyte physiology suggest that the current classification is no longer adequate and should be revised: associating one gene to one function to one tanycyte subtype is become pretty tricky, leading to confusion within the scientific community. For instance, while *β* tanycytes are associated to permeable fenestrated vessels in the ME and *α* tanycytes to BBB capillaries in the ARH, the VMH and the DMH, the permeability of vmARH vessels varies according to the energy status of the individual (10), which means that the status of tanycytes lining the vmARH would oscillate between *α* and *β* phenotypes (Figure 1). The main explanation for these issues is that intermediate zones in which tanycyte subtypes interdigitate are observed along the mature 3V ependymal layer (Figure 1) (8, 48), making difficult to reliably distinguish each subtype. Consistently, although specific characteristics and marker genes are used to separate tanycyte subtypes, many genes exhibited a gradient, rather than a clear-cut distribution across tanycyte subtypes (Figure 1). This suggests that tanycytes may be composed of continuous cell trajectories with transition zones between different subtypes and that more than four subtypes may be defined. Moreover, tanycytes belonging to a given subtype may interact with different neural cell types and different neuronal populations with a possible impact on their transcriptomic profiles resulting in different subpopulation groups within the same tanycyte subtype (Figure 1). Finally, tanycytes may also show a further degree of diversity within each subtype depending on the physiological status of the organism (10). Therefore, drawing a comprehensive picture of tanycyte molecular signature and, by this way, improving their classification is crucial and constitute the next challenge in understanding tanycyte biology and functions in the regulation of energy balance.

Major technological advances offer nowadays more powerful tools to analyze cell molecular profiles and, in our case, to improve tanycyte classification in a way that better reflects their complex biology. Recently, single-cell RNA sequencing (scRNA-seq) on dissected mediobasal hypothalamus has been used to characterize the genetic signature of hypothalamic neural cells (23, 24). Tanycyte population and their 4 subtypes have been found in these studies. Chen et al. (24) used high-throughput Drop-seq method to sequence more than 14,000 single cells obtained from dissociated hypothalamic tissues. Using semi-supervised clustering analysis, they identified 45 cell clusters with distinct gene expression signatures. Among these clusters, they distinguished one *Sox9*^+^ and *Rax*^+^ cell cluster—transcriptionally distinct from ependymocytes and other glial cell types—corresponding to tanycytes. A deeper characterization of their transcriptional heterogeneity was able to identify the four known tanycyte subtypes. In the second study, Campbell et al. (23) also used Drop-seq to analyze more than 20,000 single cells obtained from medio-basal hypothalamus (ARH-ME region), and their clustering analysis revealed two clusters for tanycytes. While data also confirm four tanycyte subtypes, they also characterize a new tanycyte gene with very restricted patterns of expression. Indeed, *Sprr1a* which participate to the impermeabilization of the skin, is found only at the border between ARH and ME, where tanycytes are thought to form a diffusion barrier (8, 49), suggesting the presence of an additional tanycyte subgroup with special diffusion barrier properties. This data therefore provides the first evidence that there is a wider range of tanycyte cell types along the third ventricle. Using tSNE map associated to *in situ* hybridization data from Allen brain atlas, these two studies defined novel markers for each tanycyte subtypes (23, 24). In these two studies, Nestin (*Nes*) and Vimentin (*Vim*) are highly transcribed in tanycytes, confirming their origin from embryonic radial glia and their function as neural stem cells in adult hypothalamus. However, these genes are also highly expressed in ependymal cells and cannot serve as tanycyte-specific markers. Some tanycyte-enriched genes found in these studies include *Col23a1, Slc16a2, Rax, Lhx2, Prdx6*, and *Ptn*. Moreover, *α* tanycyte markers include *Cd59a, Slc17a8, Crym*, and *Vcan*; α*2* and β*1* tanycyte markers include *Frzb* and *Penk;* and *β* tanycyte markers include *Col25a1, Cacna2d2*, and *Adm* genes. Additionally, *in silico* analysis of high-throughput single cell transcriptomics also allows them to define potential tanycyte functions according to their molecular signature (23, 24). Indeed, gene ontology analysis of the tanycyte-specific genes identified terms that include signal transduction, G protein-coupled receptor signaling pathway, and modulation of synaptic transmission, consistent with their known functions in transmission of metabolic information to neurons. On another hand, Campbell et al. used an analytical tool called DEPICT (Data-driven Expression Prioritized Integration for Complex Traits), designed to systematically prioritize tissue or cells based on enriched expression of GWAS-associated genes. This tool allows them to predict that transcripts from waist/hip ratio-associated loci (but not BMI, type 2 diabetes, or anorexia-linked loci) are enriched in tanycytes (23).

The first single-cell studies including tanycytes therefore brought out many new information. The novel tanycyte- and tanycyte subtype-specific markers identified will allow the development of genetic tools for delineating, labeling, and tracing the different tanycyte subtypes, as well as achieving their specific manipulation using relevant Cre or CreERT2 mouse lines in order to comprehensibly dissect their different functions in the regulation of energy metabolism. Moreover, the identification of *Sprr1a* as a specific marker for the tanycytes located at the corner of the infundibular recess confirms the existence of more than 4 tanycytes subtypes (23). However, our knowledge about tanycyte molecular signature remain basic. First, these scRNA-seq approaches have a low resolution due to the fact that they take into account many other neural cell types (only 15% of total cell number are tanycytes): this suggests that other tanycyte subtypes and specific markers are yet to be identified. Secondly, these data are still focus on the ventrodorsal organization of tanycytes but we still know very little about their anterio-posterior regionalization (43, 50). Thirdly, tanycyte molecular signature could also be impacted by neural populations with which they interact. Indeed, cells sense the presence of potential interaction partners through a wide range of receptors and, specifically respond by changing the expression of many target genes via complex regulatory networks. *α* tanycytes contacting endothelial cells, as well as different glial and neuronal populations, numerous tanycyte subtypes are consequently expected. Finally, on a wider scale, tanycytes could also be classified according to the regulatory networks to which they belong. New approaches—notably the association of tanycyte cell sorting with single cell transcriptomics, and 3D fluorescence *in situ* hybridization—are therefore necessary in order to complete tanycyte molecular classification.

## Modulation of Tanycyte Gene Expression in Response to Metabolic Challenges

Among genes expressed in tanycyte population and/or subtypes, several of them are involved in the regulation of energy balance. Although tanycytes are not unique cells expressing these genes within the metabolic hypothalamus, the functional importance of tanycyte genes is suggested by the fact that their expression is tightly regulated by the energy status and/or that their tanycyte-specific deletion have an impact on energy balance. Indeed, many studies have highlighted genes that are differentially regulated in *α* and *β* tanycytes in response to food restriction and/or, in seasonal mammals, to photoperiod (Table 1). These changes are associated with a plasticity of tanycyte function, which has been proved to be crucial to adapt the physiological response to the metabolic state and restore energy balance.

**Table 1:** Gene expression modulation in tanycytes and associated functions in the regulation of energy balance.

**Gene**	**Tanycytes**	**Functions**	**Condition**	**Regulation**
*Vegf*	β	Barrier plasticity	Fasting	Up
*Hif1*	?	Barrier plasticity	Fasting	Up
*Ocln*	α1	Barrier plasticity	Fasting	Up
*Cldn1*	β and ventral α2	Barrier plasticity	Fasting	Up
*Slc2a1*	β	Transport	Fasting	Up
*Gck*	All	Glucose sensing	Fasting	Down
*Dio2*	All	T3 bioavailibity	Long day, Fasting	Up
*Dio3*	All	T3 bioavailibity	Long day	Down
*Oatp1c1*	All	T3 bioavailibity	Long day	Up
*Slc16a2*	All	T3 bioavailibity	Long day, Fasting	Up
*Gpr50*	All	T3 bioavailibity	Long day	Up
*Nmur2*	All	T3 bioavailibity	Long day	Up
*Aldh1a1*	All	Retinoic acid signaling	Long day	Up
*Ttr*	All	Retinoic acid signaling	Long day	Up
*Crbp1*	All	Retinoic acid signaling	Long day	Up
*Stra6*	All	Retinoic acid signaling	Long day	Up
*crabp2*	All	Retinoic acid signaling	Short Day	Down
*Rarres*	All	retinoic acid signaling/Neurogenesis	Long day	Up
*rar/rxr*	All	Retinoic acid signaling/Neurogenesis	Short Day	Down
*Trhde*	β2	TRH secretion	T3 infusion	Up
*Cntf*	All	Neurogenesis	High fat diet	Up
*Fgf10*	β	Neurogenesis	Fasting	Up
*Slc1a3*	α	Neurogenesis	Short Day	Down
*Nes*	All	Neurogenesis	Short Day	Down
*Vim*	All	Neurogenesis	Short Day	Up

*Built from Langlet (3), Bolborea and Dale (4), Langlet et al. (10), Goodman and Hajihosseini (33), Salgado et al. (64), Lewis and Ebling (69), Murphy and Ebling (72), and Severi et al. (73). ? Tanycyte subtype is unknown*.

### Tanycytes Control the Access of Nutrients and Hormones Into the Metabolic Hypothalamus

To maintain energy homeostasis, tanycytes ensure an efficient communication between the periphery and the brain, notably the ARH. Indeed, *β* tanycytes form a “tanycyte barrier” by expressing tight junction proteins in a continuous belt around their cell bodies in front of fenestrated blood vessels present in the ME (8). This delocalization of barrier properties from the vascular wall to the ventricular wall gives tanycytes a key role in the control of the access for peripheral metabolites and hormones to the ARH: indeed, they constitute a “three-way exchange interface” between the blood, the CSF and the brain parenchyma (8, 10–12). During an energy imbalance, blood–ARH exchanges are crucial events to detect changes in homeostatic status and adequately answer the physiological demands. Our studies have shown that tanycytes are capable of modifying their own barrier properties to create a privileged route for circulating metabolic signals to ARH neurons (3, 51). Concretely, we observed an increase in the number of fenestrated vessels associated to the strengthening of the tanycyte barrier (10). Especially, vessels present in the vmARH, belonging to capillary loops arising from the ME, lose their usual BBB properties, and display fenestrations after 24 h fasting, allowing consequently a passive and rapid diffusion of the circulating hormones and nutrients towards a discrete population of appetite-regulating vmARH neurons (10, 52). This vascular remodeling is thought to be due to drops in blood glucose levels (10) likely detected by tanycytes themselves thanks to their glucose-sensing properties (6, 13, 14) (see below): indeed, this remodeling is mimicked by intracerebroventricular injections of non-metabolizable glucose analog in fed animals and prevented by the normalization of glucose levels in fasting animals (10). While many growth factors are involved in controlling structural plasticity in the brain, hypoglycemia-induced plasticity of the blood–ARH interface is modulated by VEGF (10), a growth factor known to induce vascular plasticity (53). It is now well-established that acute hypoglycemia rises VEGF levels, by increasing *Vegfa* mRNA expression (54), stability (55) and translation (56). In our model, transcriptional analysis on FACS-isolated tanycytes showed an increase in *Vegfa* expression specifically in tanycytes during fasting (10). Moreover, the selective knockout of *Vegfa* expression in tanycytes using a cre-lox approach revealed that the absence of tanycyte *Vegfa* regulation attenuates the effect of fasting on the ME-vmARH vascular plasticity (10). Interestingly, the up-regulation of *Vegfa* expression in tanycytes during fasting is concomitant with an increase in hypoxia-inducible factor 1α (*Hif1a*) expression, known to be involved in hypothalamic glucose-sensing (57) and to promote *Vegfa* expression (54). HIF-1a could thus be the missing link between hypoglycemia and *Vegfa* expression in our model. This vascular plasticity is accompanied by a concomitant reorganization of tanycyte tight junction complexes in both the ME and the vmARH, aiming to maintain brain homeostasis which could be disturbed by these newly permeable vessels (10). That partly results from an increase in the expression of transmembrane TJ proteins (e.g., *Ocln* and *Cldn1* mRNA) in fasting condition (10). However, further studies are necessary to determine whether the increase in TJ protein expression is directly linked to tanycyte detection of glucopenia and may be the result of the appearance of newly-fenestrated and permeable capillaries in the ME and vmARH parenchyma.

The presence of tight junction complexes at the apical pole of *β* tanycytes not only prevents the diffusion of blood-borne molecules through the paracellular cleft, but also creates cell polarity and consequently the establishment of transcytosis. In the context of energy balance, the transport of leptin (11) and ghrelin (12) towards the CSF has been observed in tanycytes. The reorganization of tanycyte tight junction complexes in conditions of energy deficit and the consecutive polarization of vmARH tanycytes may consequently impact tanycyte hormonal transports. Changes in homeostatic status also modulate nutrient transports. Indeed, gene expression analysis on FACS-isolated tanycytes in fed vs. fasted state revealed an upregulation of the facilitated glucose transporter 1 (GLUT1, also known as solute carrier family 2 *Slc2a1*) (10) in fasted condition, what may involve once again the transcription factor HIF-1 (58). If the expression of other factors involved in hormonal/nutrient transports (e.g., clathrin *Clta, Cltb* or/and *Cltc*; *lepr*) is differentially regulated by energy imbalance is still unknown.

The functional significance of the differential regulation of tanycyte genes is the opening of the “ARH window” to the periphery. Indeed, dye and hormone infusion as well as microdialysis showed an increased access of blood-borne molecules towards appetite neurons located in the vmARH (10, 52), allowing the adaptation of feeding behaviors to the nutritional status of the individual.

### Tanycytes Directly Sense the Metabolic State of the Organism

Central control of energy balance requires the monitoring of many circulating signals–including both circulating metabolites such as glucose, free fatty acids and amino acids, and secreted hormones such as ghrelin, leptin and insulin–that provide information about the nutritional status and body energy stores. Many studies have described tanycytes as metabolic sensors able to detect glucose (13, 14, 44, 46), amino acids (15), or leptin (11). Tanycytes (mainly *α* tanycytes and in a lesser extend *β* tanycytes) have mainly been shown to be able to detect changes in glucose levels in the CSF and release paracrine factors (e.g., ATP), that activate neighboring tanycytes (13) but could also potentially activate neighboring hypothalamic neurons (59, 60). The idea that tanycytes act as glucose-sensors has gained credence with the demonstration that selective glucose puffing onto tanycyte cell bodies induces Ca^2+^ waves in brain slice preparations (13) or in primary tanycyte cultures (46), as well as the immunodetection of molecules known to be essential components of glucose metabolism in pancreatic β-cells (61), such as the glucose transporter GLUT2 (62), glucokinase (63, 64), and the K_ATP_ channel subunits Kir6.1 (62, 65). However, non-metabolizable glucose analogs (e.g., 2-deoxy-D-glucose and methyl-α-D-glucopyranoside) are also capable of evoking these signals in tanycytes (13), suggesting that tanycytes would not completely mimic β-cell sensing and/or that different mechanisms exist according to tanycyte subtypes. Thereupon, three different potential mechanisms have been proposed (4), involving 1- Na^+^-linked glucose transporter (SGLT), 2- G-protein coupled receptors (Taste receptors T1r1/3 and metabotropic glutamate receptor mGluR4) (14), or/and 3- glucokinase-dependent metabolism of glucose to ATP (46, 66). The last mechanism is supported by the fact that pharmacological (46, 67) and genetic inhibition of glucokinase (*Gck*) (66) in tanycytes disturbs their glucose-sensing and have an impact on the regulation of energy balance. Interestingly, the expression and the subcellular localization of GCK varies according to the metabolic state of tanycytes. Indeed, Salgado et al. (64) have observed a 2-fold reduction of *Gck* mRNA level in the mediobasal hypothalamus of hypoglycemic rats compared to normoglycemic condition and a 2-fold increase in hyperglycemic rats. However, using tanycyte isolated by FACS in fed vs. fasted condition, we did not observe variations in *Gck* mRNA expression in mice (10). Moreover, the transcriptional regulation of *Gck* in rats is associated to a regulation of its localization and consequently of its activity (64). Hyperglycemic rats display an intense GCK nuclear localization (inactivation of GCK), whereas hypoglycemia induces a diffuse GCK immunoreaction, mainly localized in the apical pole of tanycytes (64). Future work is necessary to determine whether the expression of other components involved in tanycyte glucose-sensing is modulated in response to energy imbalance. Moreover, the different glucose-sensing mechanisms observed in tanycytes suggest that different tanycyte subtypes display differential responses to glucose, and tanycyte subgroup-specific gene expression modulation in response to glucose should consequently be investigated.

### Seasonal Cycles Modulate Tanycyte Functions

The regulation of gene expression in tanycytes to control energy metabolism has been largely documented in the context of seasonal cycles (4, 68, 69). In the natural environment, mammalian models sensitive to photoperiod (e.g., CBA/N and C3H mice, F344 rats, Djungarian hamsters) adopt behavioral and physiological adaptations (i.e., hibernation, daily torpor, migration, changes in pelage, reproduction, and altered feeding) resulting from both innate rhythmical processes orchestrated by photoperiod and an adaptation to food availability. In general, mammals increase food intake and accumulate energy stores in spring and summer (corresponding to a long photoperiod); and then reduce appetite, conserve energy by entering in hypometabolic states and/or survive by catabolizing their stored energy depots in winter (corresponding to a short photoperiod). Therefore, seasonal animals are useful models for studying differential patterns of gene expression related to energy expenditure and appetite. Interestingly, numerous genes expressed in tanycytes display a photoperiodic regulation of their expression, confirming a crucial role of these cells in the control of energy balance. These changes in gene expression mainly include gene involved in thyroid hormone signaling (e.g., *Dio2, Dio3*, and *Oatp1c1*), and retinoic acid signaling (e.g., *Raldh1, Crbp1, Ttr*, and *Stra6*) pathways (4, 69).

First, the photoperiodic control of metabolism by tanycytes mainly relies on their ability to locally regulate thyroid hormone bioavailability in the metabolic hypothalamus through the regulation of deiodinases (*Dio2* and *Dio3*) expression (72, 73). Thyroid hormone (triiodothyronine or T_3_) is a regulator of energy balance and lipid metabolism, though peripheral and central effects (74): many studies have shown that a decrease in T_3_ reduces food intake and promotes the catabolism of abdominal adipose tissue, whereas T_3_ hypothalamic infusion inhibit the reduction of appetite and loss of weight that normally occurs under short photoperiod (70). Moreover, T_3_ regulates the responses of neuropeptide Y (NPY)-containing neurons in the arcuate nucleus to food deprivation (30). Initially synthesized as a prohormone, L-thyroxine (or T_4_) is converted by DIO1 and 2 to the active hormone, triiodothyronine (or T_3_), which can be then inactivated by DIO3 to the inactive form called T_2_. In the mediobasal hypothalamus, tanycytes are the main locus for *Dio2* and *Dio3* expression (72, 73). They also express the organic anion transporting polypeptide 1C1 (*Oatp1c1*) and monocarboxylate transporter 8 (MCT8, *Slc16a2* gene) (75), which are involved in the uptake of T_4_ and T_3_. The hypothesis is that the prohormone T_4_ is taken up by tanycytes from the circulation or the CSF via MCT8 and OATP1C1, DIO2 then converts T_4_ to the active T_3_, which can diffuse into the surrounding hypothalamic nuclei and act on neurons involved in the regulation of metabolism (76). Interestingly, these genes are differently regulated in tanycytes. One of the most profound changes in *Dio2* and *Dio3* expression occur in numerous seasonal rodents: under short photoperiod, a downregulation of *Dio2* and upregulation *Dio3* have been observed in hamster and rat tanycytes (77, 78), leading to a decrease in the bioavailability of T_3_ in the hypothalamus and consequently in a decrease of appetite (68). Moreover, tanycytes may also integrate a number of other signals in addition to photoperiodic information in order to regulate hypothalamic thyroid hormone bioavailability. For example, food deprivation increases *Dio2* and *Slc16a2* mRNA in *β* tanycytes of rats, potentially allowing a global increase in T_3_ levels in the hypothalamus to stimulate food intake (79, 80). Different mechanisms have been proposed to explain these variations in gene expression. The main neuroendocrine mechanisms underlying these metabolic changes rely on the regulation of melatonin secretion by the pineal gland according to changes in the nocturnal duration (81). Indeed, different studies showed that changes in melatonin secretion alter the release of paracrine factors from the pars tuberalis, which in turn regulates gene expression in tanycytes, notably those encoding DIO enzymes (82). Different paracrine factors modulating tanycyte gene expression have been described including the b subunit of thyroid stimulating hormone (*Tshb*) (83), and the neuropeptide Neuromedin U (*Nmu*) (84). TSH receptor and NMU receptor are expressed in tanycytes and their activation induce *Dio2* expression (85). Interestingly, NMU receptor (*Mnur2*) are photoperiodically regulated in the hypothalamus of F344 rats, with a highest level during long days (86). Some other receptors have also been shown to be expressed in tanycytes, differently regulated according the day length, and involved in the regulation of *Dio2* expression. A first candidate is FGFR1c, a receptor for a family of growth/endocrine factors (including FGF2 and FGF21), involved in the regulation of energy homeostasis (87) and expressed in tanycytes (88). The selective inhibition of this receptor by local infusion of neutralizing antibodies into the third ventricle of Siberian hamsters reduced food intake and body weight as well as *Dio2* expression in tanycytes during long days (when *Dio2* expression is normally high and animals gain weight), but not during short days (when *Dio2* expression is low and animals lose weight) (89). GPR50, a receptor having homology with the melatonin receptors although it does not bind melatonin, is also functionally linked to seasonal metabolic regulation, especially in the context of adaptive thermogenesis and torpor (90). Present in tanycytes (91, 92), *Gpr50* expression is downregulated in the Djungarian hamster under short photoperiods (93), when these animals are prone to display torpor. Moreover, *Gpr50* knockout (KO) mice display a state of torpor when fasted or treated with 2-deoxyglucose (90): this effect appear to be mediated via thyrotropin-releasing hormone (TRH) given that it is reversed by treatment with TRH receptor agonists (90). Interestingly, tanycyte *Dio2* expression was constitutively elevated in fed *Gpr50* KO mice (90), suggesting that GPR50 indirectly modulates T3 handling in tanycytes what may therefore influence thermogenesis (74). Additionally, thioredoxin-interacting protein (*Txnip*) expression is induced in tanycytes of *Gpr50* KO mice during fasting, what may be critical to regulate energy expenditure and fuel use, and may consequently induce a torpid state (94). Besides the role of GPR50 during fasting, GPR50 KO mice are also resistant to a high-fat diet, suggesting a role in metabolic regulation (95). While further studies are needed to confirm that, this hypothesis is reinforced by the fact that GPR50 significantly alters transcriptional responses to leptin signaling (90) and that TXNIP specifically regulates leptin sensitivity in NPY neurons (96).

Besides thyroid hormone signaling, the retinoic acid signaling pathway in tanycytes also regulates seasonal metabolic changes. Indeed, there is extensive evidence in both hamsters and F344 rats that transporters, binding proteins and synthetic enzymes involved in this pathway display seasonal alterations of expression in tanycytes (97, 98). For instance, the expression of the enzymes synthesizing retinoic acid (retinaldehyde dehydrogenase 1 and 2, *Raldh1*, and *Raldh2*) is reduced in tanycyte F344/N rats during short days, and this process is reversed by treatment with thyroid hormone (99). Furthermore, transporters for retinoic acid including retinoic acid gene 6 homolog (*Stra6*), transthyretin (*Ttr*), and cellular retinoic acid binding protein 1 (*Crbp1*), are downregulated in tanycytes of F344 rats and Siberian hamsters under short photoperiod (86, 93, 98). Interestingly, these changes are blocked by pinealectomy, highlighting the importance of melatonin in this process. This transcriptional regulation of genes involved in retinoic acid signaling is potentially highly significant given that retinoic acid regulates tanycyte proliferation and their ability to generate new cells in the hypothalamus.

Collectively, these studies show that tanycytes respond to photoperiodic information and to nutritional information by modulating genes involved in thyroid hormone and retinoic acid signaling pathway to modulate their own function but also the activity of neighboring appetite-regulating neurons.

### Tanycytes Control TRH Neurosecretion

The ME has been primarily described as a neurosecretory circumventricular organ. Indeed, it contains neurosecretory axons that either travel towards the neurohypophysis in order to release their contents into the general circulation or reach the ME fenestrated vessels to deliver their neurohormones into the hypothalamo-hypophysial portal system. Whereas, β*1* tanycytes dynamically interact with the axon terminals of the GnRH neurons that control the hypothalamic-pituitary-gonadal axis (5, 27), β*2* tanycytes interact with other neuroendocrine neuronal populations, in particular with the terminals of TRH neurons that control the hypothalamic-pituitary-thyroid axis (76), suggesting that tanycytes may play a pivotal role in the control of TRH release.

TRH is released in the pituitary portal circulation and then targets thyrotrope cells in the anterior pituitary to stimulate the secretion of thyroid-stimulating hormone (TSH). In turn, TSH stimulates the thyroid gland to synthesize and secrete the thyroid hormone T_4_, that will be converted to T_3_ by DIO1 and DIO2 to be active (see previous paragraph). Interestingly, tanycytes modulate TRH secretion by, at least, three different ways. First, tanycytes being the main mediators of the DIO2-depending conversion of T_4_ to T_3_ within the mediobasal hypothalamus, T_3_ released by tanycyte endfeet would be taken up by neighboring TRH axon terminals and retrogradely transported to their cell bodies in the PVH (100) to inhibit TRH transcription (101, 102). Therefore, the modulation of *Dio2* expression in tanycytes during energy imbalance or seasonal cycles may influence TRH transcription. Secondly, tanycytes express TRH-degrading ectoenzyme (*Trhde*, pyroglutamyl peptidase II), an enzyme that inactivates TRH in the extracellular space, suggesting that tanycytes could directly regulate TRH levels before its passage into the pituitary portal circulation (103). Interestingly, the expression of *Trhde* in β*2* tanycytes is upregulated following systemic administrations of T_4_ (103), forming a negative feedback loop to control the circulating levels of TRH. *Trhde* expression is also upregulated during fasting, leading to the downregulation of the hypothalamus-pituitary-thyroid axis in this metabolic state (104). Finally, the activation of TRH receptor 1 increases intracellular calcium in *β* tanycytes through Gα_q/11_ proteins, leading to the outgrowth of the tanycyte processes enwrapping TRH neuroendocrine terminals, and an upregulation of the activity of TRHDE, limiting TRH release into the pituitary portal circulation (28). However, if this calcium signaling modulates *Trhde* expression has not been investigated.

### Tanycytes Act as Neural Stem Cells in Response to Dietary Cues

Tanycytes, regarded as putative remaining radial glial cells in the adult brain, have maintained their capacity to proliferate in the postnatal brain and in a lesser extend in the adult brain. Indeed, many studies showed that *α* and *β* tanycytes act as progenitor cells—*in vivo* and *in vitro*—able to differentiate into both neurons and glia, including astrocytes and other tanycytes (4, 32, 33).

As mentioned previously, tanycytes express a variety of neural stem/progenitor cell markers (17, 40, 47, 105–109) such as *Nes, Vim, Sox2, Fabp7, Slc1a3, Musashi-1, Gfap, Notch1, Notch2, Hes5, Lhx2, Rax*, UGS148, and *Prss56*. Interestingly, tanycytes are heterogeneous with regard to the expression of these progenitor cell markers, their proliferative capacities, and the fate of their progeny. For instance, *α* tanycytes mainly proliferate to renew part of the tanycyte population, and generate astrocytes and a few neurons (40, 110), whereas FGF10+ tanycytes (corresponding to *β* tanycytes) mainly produce neurons (17). This heterogeneity also occurs over time: young postnatal tanycytes give birth to neurons that are mostly found in the ME (16), whereas adult tanycytes generate neurons and, to a lesser extent, glial cells that are mostly found in the arcuate, ventromedial, dorsomedial, lateral and posterior nuclei (110). These differences, which result from the different transcriptomic profile between tanycyte subtypes, may be due to a different embryological origin. Indeed, tanycytes lining the lateral walls of the ventricle derive from the sonic hedgehog (*Shh*)-expressing floor-plate, and retain *Shh* expression in adulthood (111). *Shh* exerts numerous actions during the development of the central nervous system, ranging from proliferation to cell fate of new born cells (112). Therefore, better characterization of the molecular heterogeneity of tanycytes is more than ever necessary to clarify our understanding of the complexity of the hypothalamic niche.

The link between this proliferative capacity and energy balance is mainly based on the hypothesis that tanycytes could contribute to the plasticity and remodeling of hypothalamic neural (including tanycytes, astrocytes, and neurons) networks controlling energy balance. Indeed, neurons born from *β* tanycytes during the early postnatal period respond to fasting with an increased in c-fos expression (16). Another study showed that neurons born from *β* tanycytes during the prepubertal period respond to leptin administration by the phosphorylation of STAT3 in the ARH (17). Moreover, studies have shown that blocking hypothalamic neurogenesis (37, 115) induces obesity, suggesting its important role for the control of energy balance. However, a contrasting study showed that β*2* tanycytes increased their proliferation in young mice under high-fat diet, and that blocking neurogenesis in ME is protective against high-fat diet induced weight gain (16). These differences may once again result from different mechanisms observed in different tanycyte subtypes, or the implication of sex-specific factors (116). Alternatively, these differences may also be explained by the fact that neural stem cells other than tanycytes are present in the mediobasal hypothalamus and involved in the regulation of metabolism (37, 115).

The adaptive response of tanycyte neurogenesis to energy imbalance to induce neural network plasticity may involve the modulation of tanycyte neural stem cell marker expression. Ciliary neurotrophic factor (*Cntf* ) and its receptor (*Cntfr*) are known to stimulate neurogenesis in hypothalamic feeding centers, yielding leptin-responsive NPY and POMC neurons and a reduction of food intake and body weight (117). Interestingly, *Cntf* and *Cntfr* are mainly expressed by ependymal cells and tanycytes in the hypothalamus (42), and their expression is upregulated in response to high fat diet (71).

Another physiological context that affects hypothalamic neurogenesis is seasonality. In sheep, the Sox2-expressing tanycyte layer appears thicker during short photoperiod and hypothalamic cell proliferation is observed (118, 119). Moreover, the expression of neural stem cell markers including *Nes, Vim, Gfap*, and *Dcx* increase in the hypothalamus compared to long photoperiod (118, 119), confirming an increase in neurogenesis. T_3_ and retinoic acid being modulated by photoperiod and able to modulate neurogenesis (120, 121), whether their bioavailability affects adult hypothalamic neurogenesis in relation to feeding is an important question for future research.

### Perspectives Regarding Gene Expression Dynamics in Tanycytes

Many questions are pending regarding tanycyte gene expression dynamics in response to energy imbalance. What other genes are modulated in the regulation of tanycyte functions? What are the consequences of tanycyte gene expression modulation on neuronal activity? A comprehensive list of modulated genes would be useful to fully understand the role of tanycyte in the reestablishment of energy homeostasis. Although some studies begin to focus on it—such as the two scRNAseq studies described previously where the authors reveal energy status-sensitive populations (23, 24)–, further works will be necessary to decipher tanycyte gene expression dynamics and their consequences in the regulation of energy imbalance.

## Molecular Mechanisms Underlying Gene Expression Dynamics in Tanycytes

### How to Modulate Gene Expression?

Gene expression is a multistep process that involves gene transcription (e.g., chromatin remodeling, transcription factors, and co-regulators), mRNA processing (e.g., capping, splicing, and polyadenylation), mRNA degradation, transport and translation (e.g., RNA interference, RNA-binding proteins). Each of these processes is controlled by a complex series of biochemical events occurring in different locations within the cell, as well-illustrated in the literature (122–124). Despite that, transcriptional regulation, and in particular the control of transcriptional initiation, constitutes the primary regulation site, and much attention has been focused on this process (122).

**Figure 2:**
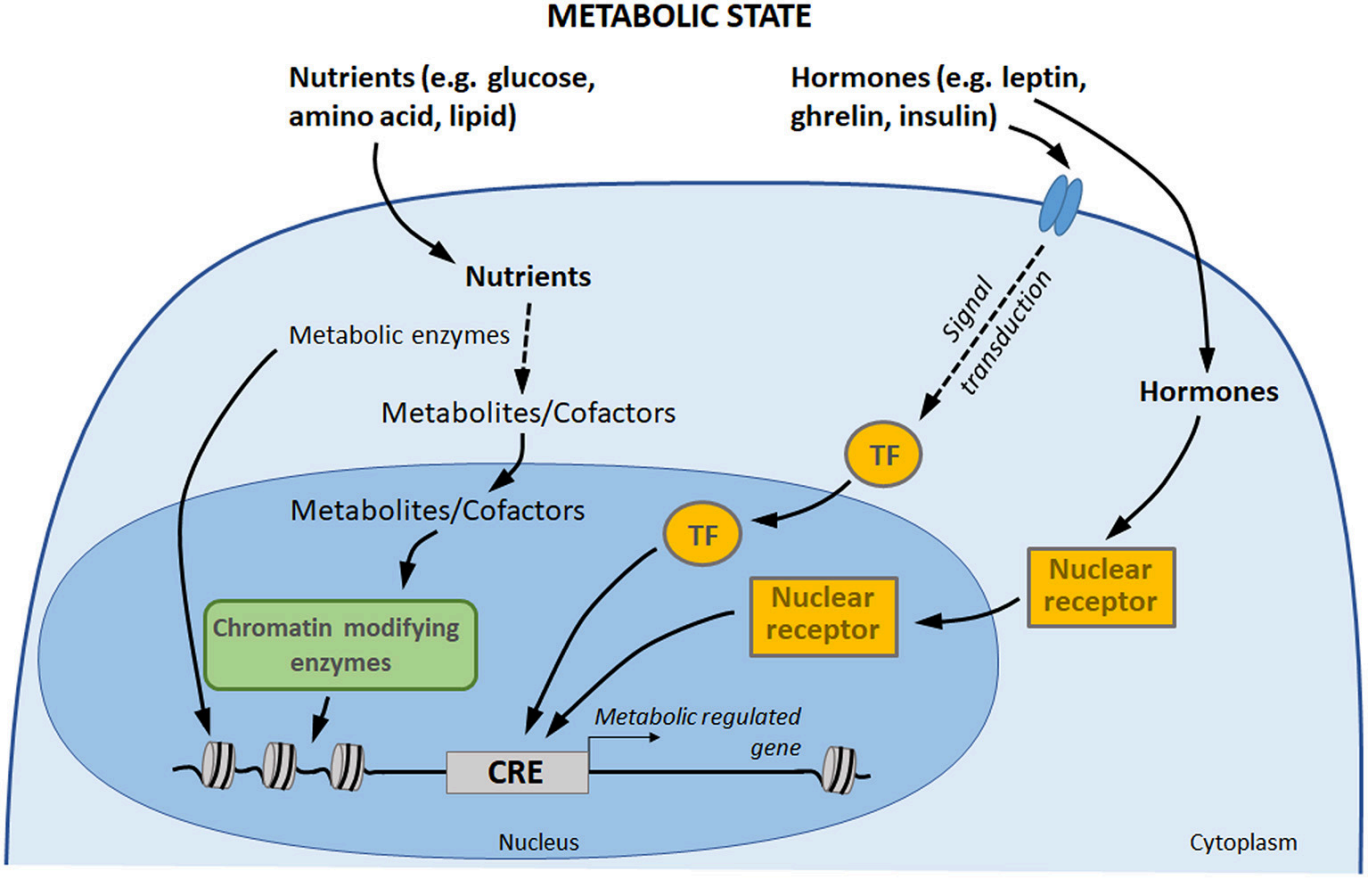
Gene expression control by hormones and nutrients. Transcription factors and nuclear receptor are activated by extracellular signals, such as hormones, which may directly induce a transcriptional response to changes in metabolic state. In addition, nutrients are metabolized by the cells. Some components of intermediary metabolism are cofactors or co-substrates of chromatin-modifying enzymes, which may affect chromatin structure and gene expression. Finally, some metabolic enzymes also act as regulators of chromatin and transcription factors (113, 114). TF, transcription factors; CRE, cis-regulatory element.

Metabolic circulating factors, including hormones and nutrients, are able to influence several processes in organisms, including gene expression (18, 125). By impacting gene expression in different tissues, metabolic factors allow the organism to acclimate to its new environment and to ensure energy homeostasis (126). There is a growing awareness for a direct involvement of these metabolic signals in transcriptional regulation control, through three main processes (Figure 2) (127, 128). (1) Nutrients and hormones (e.g., leptin, ghrelin, insulin, glucose) are able to activate signaling pathway leading to the binding of specific transcription factors to specific DNA sequences in order to initiate transcription. (2) On another hand, vitamins, hormones, and metabolites (e.g., steroid hormones, thyroid hormones, retinoic acid, and vitamin D_3_) can directly influence gene transcription by binding nuclear receptors: though this direct pathway, the receptor itself acts as a transcription factor. (3) Finally, central components of nutrient intermediary metabolism (e.g., acetyl-CoA) are cofactors or co-substrates of chromatin-modifying enzymes (e.g., histone deacytylases, methyltransferases, acetyltransferases): their concentrations therefore constitute a potential regulatory interface between the metabolic and chromatin states.

Although the regulation of gene expression by nutrients and hormones is well-documented in numerous peripheral tissues, such molecular mechanisms in tanycytes are poorly described.

### Signaling Pathways Modulated by Hormones and Nutrients in Tanycytes

Being at the interface between the blood and the brain, tanycytes are in a privileged position to integrate multiple metabolic inputs which could modulate their gene expression. Indeed, multiple studies described tanycytes as sensors of these metabolic circulating factors, including glucose, amino acids, leptin, and ghrelin. Several studies have shown that these metabolic factors are able to activate different signaling pathways in tanycytes.

Leptin mediates its effect through the activation of several signaling pathways, including Januskinase/Signal transducer and activator of transcription (JAK/STAT) (129). This signaling cascade leads to phosphorylation of the STAT3 transcription factor, which dimerizes and translocates to the nucleus where it regulates the expression of a variety of genes involved in cell growth, angiogenesis, or inflammation (130, 131). For instance, activation of STAT3 trans-activates *Vegfa* promoter and increases *Vegfa* expression through HIF1 transcription factor (132, 133). In the hypothalamus, and in particular in tanycytes, JAK/STAT pathway serves as the primary leptin signal transduction pathway (129, 130). *In vitro*, tanycytes express a number of splice variants of ObR, and treatment with leptin result in activation of some signaling pathways (e.g., phosphorylation of AKT, STAT3, and ERK) (11). *In vivo*, leptin-induced pSTAT3 immunoreactivity first appears in *β* tanycyte processes contacting ME fenestrated vessel, and then their cell nuclei located close to the floor of the third ventricle (11). Tanycyte STAT3 signaling is also activated by ciliary neurotrophic factor (CNTF) (42), a factor known to cause weight loss in obese rodents and human through leptin-like activation of the Jak/STAT3 signaling pathway (134).

MAPK/ERK signaling which can be induced by leptin and ghrelin is also active in tanycytes (11, 12). It has been shown that activating ERK pathway through EGF treatment allows the liberation of tanycyte-endocytosed leptin and the restoration of its central anorectic effect (11). TSH also increases ERK phosphorylation in primary tanycyte cultures (135).

As other glial cells, calcium signaling is crucial in tanycytes. Induced by different stimuli including glucose (13), amino acids (15), ATP (13), non-metabolizable glucose analogs (14), TRH (28), the increase in intracellular calcium in tanycytes is able to propagate as calcium waves from one tanycyte to another through gap junctions (13, 44). These calcium waves may synchronize gene expression in tanycytes and/or tanycyte subgroups. While these different signaling pathways involved in the control of energy balance are present in tanycytes, nothing is so far known about the consequences of their activation on tanycyte or tanycyte subtype-specific gene expression.

### Transcription Factors Mobilized in Tanycytes

Recent findings show that the specification and differentiation of hypothalamic tanycytes during development is partly controlled by LHX2 and RAX transcription factors (107, 136). LHX2 and RAX transcription factors are both expressed in the developing hypothalamus and maintained in adult tanycytes (107, 137). Following the embryonic deletion of *Lhx2*, ependymal cells lining the floor of the third ventricle -presumptively tanycytes- exhibit a hybrid ependymal cell/tanycyte identity (107). In particular, they lose the tanycyte-specific expression of *Rax*, and display an ectopic expression of cuboid ependymal cells-specific *Rarres2* (107). Moreover, they retain radial morphology while becoming multiciliated. In contrast, postnatal loss of function of *Lhx2* results only in loss of tanycyte-specific gene expression (107). If these transcription factors play a role in tanycyte neurogenesis in response to energy imbalance is still unknown.

In the context of seasonal cycles, the expression of nuclear transcription factor NF-κB (nuclear factor kappa-light-chain-enhancer of activated B cells) has been shown to be decreased in rat tanycytes under long photoperiod, which could modulate the expression of numerous inflammatory genes (138).

### Epigenetic Regulations in Tanycytes

The structural state of chromatin is another critical point in gene expression regulation. Wrapped around eight histone protein cores, DNA can be tightly packed, leading to gene repression. In contrast, decondensed chromatin makes DNA accessible to the transcriptional machinery, promoting gene expression. Fluctuations between open and closed chromatin partly occur through histone modifications (e.g., acetylation, methylation, ubiquitination). In the context of energy balance, metabolites are cofactors or co-substrates of histone-modifying enzymes affecting by this way gene expression. Some metabolic enzymes also act as regulators of chromatin [see review (113, 138)]. In photoperiod-sensitive F344 rats, histone deacetylase 4 (*Hdac4*, a class of chromatin modifying enzymes) expression increases in tanycytes during long days, partly due to TSH stimulation. Interestingly, specific inhibitors showed that HDAC4 represses target genes of NF-κB and thyroid hormone receptor, that could limit inflammation and thyroid action in the hypothalamus during long days (138).

### Perspectives Regarding Gene Expression Regulation in Tanycytes

What are the transcription factors, nuclear receptors and co-substrates of chromatin-modifying enzymes involved in the modulation of tanycyte gene expression? Which other stimuli may influence tanycyte gene expression and functions? Besides the initiation of transcription, which steps in gene expression process (e.g., translation) are modulated by energy imbalance in tanycytes? Many questions remain to be answered but science now offers more powerful techniques which will allow considerable progress in this area. In particular, chromatin remodeling, DNA accessibility and non-coding RNA are now measurable on FACS-isolated tanycytes. To continue research efforts in this direction will help to address many of the unresolved questions concerning tanycyte functions and how they may relate to human health and disease.

## Conclusion Remarks

It is now well-established that tanycytes and tanycyte subgroups play diverse, yet complimentary, metabolic functions, ranging from sensing, shuttling, and release of nutrients and hormones within the hypothalamus, in order to influence neural appetite networks. Displaying a huge molecular and functional heterogeneity, the exhaustive elucidation of their different molecular signature will help our understanding of these diverse physiological functions. Recent advances in gene expression profiling opened a new research area where there is much to learn in the future.

Moreover, the study of nutrients and hormones as regulators of gene expression in tanycytes is clearly a key field to dig in order to fully understand their role in the regulation of energy metabolism. Although such regulation is well-documented in peripheral tissues, it stays poorly described in the brain, and in particular in tanycytes. The impact of metabolic signals on gene transcription is likely not involved in short-term control (i.e., seconds to minutes), but rather in longer-term adaptive responses (i.e., hours to days). By changing the expression of key proteins that are involved in tanycyte metabolic function and cellular processes such as metabolism, these molecular modulations would allow tanycytes to face changes in nutritional status and to adequately respond to them.

Ultimately, genetic manipulation of tanycyte function will offer a helpful tool for modulating energy balance in order to tackle eating disorders such as obesity and anorexia.

## Author Contributions

The author confirms being the sole contributor of this work and has approved it for publication.

### Conflict of Interest Statement

The author declares that the research was conducted in the absence of any commercial or financial relationships that could be construed as a potential conflict of interest.
